# Decreased CX3CR1 messenger RNA expression is an independent molecular biomarker of early and late mortality in critically ill patients

**DOI:** 10.1186/s13054-016-1362-x

**Published:** 2016-06-30

**Authors:** Arnaud Friggeri, Marie-Angélique Cazalis, Alexandre Pachot, Martin Cour, Laurent Argaud, Bernard Allaouchiche, Bernard Floccard, Zoé Schmitt, Olivier Martin, Thomas Rimmelé, Oriane Fontaine-Kesteloot, Mathieu Page, Vincent Piriou, Julien Bohé, Guillaume Monneret, Stéphane Morisset, Julien Textoris, Hélène Vallin, Sophie Blein, Delphine Maucort-Boulch, Alain Lepape, Fabienne Venet, for the MIP Rea Study Group, Guillaume Marcotte, Christian Guillaume, Romain Hernu, Sylvie De La Salle, Marie Simon, Thomas Baudry, Elisabeth Cerrato, Emmanuelle Gallet-Gorius, Audrey Larue, Christine Alberti-Segui, Nathalie Panel, Marion Provent, Jean-Paul Viale, Anne Portier

**Affiliations:** Hospices Civils de Lyon, Intensive Care Unit, Centre Hospitalier Lyon Sud, Pierre Bénite, France; Hospices Civils de Lyon-bioMérieux Joint Research Unit, Groupement Hospitalier Edouard Herriot, Lyon, France; Hospices Civils de Lyon, Medical Intensive Care Unit, Groupement Hospitalier Edouard Herriot, Lyon, France; Hospices Civils de Lyon, Department of Anaesthesiology and Critical Care Medicine, Groupement Hospitalier Edouard Herriot, University Claude Bernard Lyon 1, Lyon, France; Hospices Civils de Lyon, Intensive Care Unit, Hôpital de la Croix Rousse, Lyon, France; Hospices Civils de Lyon, Immunology Laboratory, Groupement Hospitalier Edouard Herriot, Lyon, France; Hospices Civils de Lyon, Université Lyon 1, CNRS, UMR5558, Service de Biostatistique et Laboratoire de Biométrie et Biologie Evolutive, Equipe Biostatistique-Santé, Lyon, France; Immunology Laboratory, Hôpital E. Herriot - Hospices Civils de Lyon, 5 place d’Arsonval, 69437 Lyon Cedex 03, France

**Keywords:** CX3CR1 mRNA, Transcriptomics, Biomarker, Mortality, Intensive care unit, Prognosis, Immunosuppression

## Abstract

**Background:**

Chemokine (C-X3-C motif) receptor 1 (CX3CR1) was identified as the most differentially expressed gene between survivors and non-survivors in two independent cohorts of septic shock patients and was proposed as a marker of sepsis-induced immunosuppression. Whether such a biomarker is associated with mortality in the heterogeneous group of critically ill patients is unknown. The primary objective of this study was to evaluate the association between CX3CR1 messenger RNA (mRNA) expression and mortality in intensive care unit (ICU) patients. The secondary objective was to evaluate similar endpoints in the subgroup of septic shock patients.

**Methods:**

We performed a prospective, multicentre, non-interventional study in six ICUs of university hospitals in Lyon, France. Every consecutive adult patient with systemic inflammatory response syndrome and an expected length of stay in the ICU over 2 days was included. Whole-blood CX3CR1 mRNA expression was measured by quantitative real-time polymerase chain reaction at day 1 (D1) and D3 after inclusion.

**Results:**

In ICU patients (*n* = 725), decreased CX3CR1 mRNA expression at D1 was associated with high D7 mortality (AUC 0.70, adjusted OR [aOR] 2.03, 95 % CI 1.19–3.46), while decreased expression at D3 was associated with increased D28 mortality (AUC 0.64, aOR 2.34, 95 % CI 1.45–3.77). In septic shock patients (*n* = 279), similar associations were observed between decreased D1 CX3CR1 mRNA expression and D7 mortality (AUC 0.69, aOR 2.76, 95 % CI 1.32–5.75) as well as decreased D3 expression and D28 mortality (AUC 0.72, aOR 3.98, 95 % CI 1.72–9.23). These associations were independent of lactacidaemia, Simplified Acute Physiology Score II, Sepsis-related Organ Failure Assessment score and Charlson comorbidity index.

**Conclusions:**

This study represents the largest evaluation of such an mRNA marker in a heterogeneous cohort of severely injured patients. Our results show that decreased CX3CR1 mRNA expression is associated with increased mortality in ICU patients. This suggests a link between injury-induced immunosuppression and mortality in critically ill patients. In this context, the monitoring of such a host response molecular biomarker could prove very helpful for the identification of patients at high risk of death in the ICU.

**Electronic supplementary material:**

The online version of this article (doi:10.1186/s13054-016-1362-x) contains supplementary material, which is available to authorized users.

## Background

Intensive care units (ICUs) treat a heterogeneous group of patients with life-threatening injuries requiring close monitoring and support with high-technology medical equipment and multiple medications. In this context, the search for prognostic biomarkers that would help clinicians predict patients’ outcomes has been ongoing for years [1].

Several aspects of risk stratification biomarkers could be of interest in the ICU, especially in the context of financial constraints faced by the health care system and the strong need for optimization of limited medical resources. Indeed, besides use as benchmarking tools and in inter-ICU comparative studies, accurate outcome prediction based on biomarkers could be extremely useful for improving clinical decision-making. For example, biomarkers could help clinicians to dispense relevant treatments to patients who might benefit from them [2].

Transcriptomics has recently emerged as a novel tool to identify such biomarkers [3–8]. Most important, the availability of fully automated and user-friendly molecular platforms offers concrete solutions for routine clinical applications, even in ICU and emergency settings.

We previously identified chemokine (C-X3-C motif) receptor 1 (*CX3CR1*) as the most differentially expressed gene between survivors and non-survivors after septic shock [9, 10]. CX3CR1 is the sole receptor for fractalkine (CX3CL1) expressed on leucocyte subpopulations. Interestingly, we observed an association between decreased CX3CR1 expression on monocytes after septic shock and decreased functional responses of monocytes ex vivo [10]. This provides a link between decreased CX3CR1 and immune dysfunction after sepsis and suggests that the measure of such a host response messenger RNA (mRNA) marker may be useful in evaluation of injury-induced immune alterations. However, whether such a biomarker could be useful in the heterogeneous group of ICU patients is unknown. Therefore, we conducted a prospective, multicentre, non-interventional study with the primary objective of studying the association between CX3CR1 mRNA expression and risk of death in a large cohort of critically ill patients. The secondary objective was to evaluate the association between CX3CR1 mRNA expression and risk of death in the subgroup of septic shock patients.

## Methods

### Experimental design

This prospective, multicentre, non-interventional study was conducted in six ICUs in Lyon, France. It was approved by our institutional ethical review board (Comité d’Ethique des Centres d’investigation Clinique de l’Inter-Région Rhône-Alpes Auvergne – IRB 5044).

### Follow-up and outcomes

The primary outcome was the association of day 1 (D1) and D3 CX3CR1 mRNA expression and risk of death during the hospital stay of ICU patients with systemic inflammatory response syndrome (SIRS). The secondary outcome was the association of D1 and D3 CX3CR1 mRNA expression and risk of death during the hospital stay in the subgroup of septic shock patients. In both cases, mortality was evaluated at 7 days (D7) and at 28 days (D28).

Since half of the deaths in our cohort occurred before D7 and the median ICU stay was 7 days, this time point (i.e., D7) was used to define early mortality among the 725 patients eligible at D1. Conversely, D28 was used to define late mortality after ICU admission among the 515 patients eligible at D3.

### Patients

From December 2009 to June 2011, every consecutive patient aged ≥18 years with an expected length of stay in the ICU of more than 2 days was prospectively enrolled if the patient met the criteria for SIRS as described in the American College of Chest Physicians/Society of Critical Care Medicine (ACCP/SCCM) 1992 consensus statement [11] and if non-opposition to inclusion in the protocol was obtained.

Exclusion criteria comprised immunosuppression as defined by immunosuppressive treatment or corticoid treatment with dosage >10 mg/day or cumulative dose >700 mg equivalent prednisolone, aplasia as defined by number of circulating neutrophils <500 cells/mm^3^, primary innate immune deficiency, and extracorporeal circulation during the month before ICU admission.

No informed consent was needed, as this study was non-interventional and complementary blood samples in PAXgene® tubes (PreAnalytiX, Hombrechtikon, Switzerland) were obtained during patients’ routine blood sampling after completion of routine follow-up tests. Nevertheless, non-opposition to inclusion in the protocol was recorded from every patient or the patient’s next of kin.

### Definitions

SIRS was defined as the presence of at least two of the following clinical criteria: temperature >38 °C or <36 °C, heart rate >90 beats/minute, respiratory rate >20 breaths per minute or PaCO_2_ < 32 mmHg (4.3 kPa), and leucocyte count >12,000/mm^3^ or <4000/mm^3^ [11].

Sepsis was defined as the presence of a proven (visible either clinically/surgically, radiologically or microbiologically) infection or a highly suspected infection at inclusion. Following the definitions proposed by Vincent et al., which partly rule out the former definitions of sepsis and severe sepsis described in the ACCP/SCCM 1992 consensus conference statement, sepsis was simply defined as an infection requiring ICU admission [12].

Shock was defined as persistent hypotension despite adequate fluid resuscitation requiring the use of epinephrine or norepinephrine at a dose >0.25 μg/kg/minute [13].

Antibiotic treatment was considered to be appropriate if all the pathogenic microorganisms were sensitive to at least one of the administered antimicrobial drugs as determined by an in vitro sensitivity pattern. In cases of culture-negative infection, treatment was considered appropriate if consistent with the local anti-infectious protocols.

### Data collection at inclusion

The following demographic, clinical and biochemical data were collected at patient admission and/or inclusion in the protocol:

1. Demographic characteristics: age and sex
2. Clinical scores: Simplified Acute Physiology Score II (SAPS II) (range 0–163 [14], calculated at inclusion), Sepsis-related Organ Failure Assessment (SOFA) score (range 0–24 [15], calculated at inclusion and at D3) and Charlson comorbidity index (range 0–32 [16] for which age was not taken into account).
3. Lactic acid concentration (at D1 and at D3)
4. Need for respiratory (mechanical ventilation), haemodynamic (vasopressor treatment) or renal (renal replacement therapy) support
5. Presence of shock (defined as a cardiovascular SOFA score of 4)
6. Diagnostic category (planned or urgent, surgery or medical)
7. Presence of trauma
8. Presence of sepsis (either community- or hospital-acquired)
9. Characteristics of infection if relevant (site, type of detection, identified germs)
10. Presence of an anti-infectious treatment if relevant and its adequacy either to the antibiogram or to the local anti-infectious protocol

### RNA extraction, reverse transcription and quantitative polymerase chain reaction

PAXgene® tubes were collected from patients within the first 12 h after inclusion in the protocol (D1) and at D3. Total RNA was extracted using the PAXgene® Blood RNA Kit (PreAnalytiX). Before RNA elution, the residual genomic DNA was digested using the RNase-Free DNase Set (QIAGEN, Hilden, Germany). Total RNA was reverse-transcribed into complementary DNA (cDNA) using the SuperScript VILO cDNA Synthesis Kit (Life Technologies, Carlsbad, CA, USA). The candidate gene was quantified using quantitative real-time polymerase chain reactions. Polymerase chain reactions (PCRs) were performed in a LightCycler instrument (Roche Diagnostics, Risch-Rotkreuz, Switzerland) using the standard TaqMan Fast Advanced Master Mix PCR kit according to the manufacturer’s instructions (Applied Biosystems, Foster City, CA, USA). Thermocycling was performed in a final volume of 20 μl containing 5 μM of required primers and 1 μM of required probe. PCR was performed with an initial denaturation step of 10 minutes at 95 °C, followed by 45 cycles of a touch-down PCR protocol (10 seconds at 95 °C, 29 seconds of annealing at 68 °C and a 1-second extension at 72 °C). The cDNA standards were prepared from purified PCR amplicons obtained with the corresponding primers (Additional file 1: Table S1). The second derivative maximum method was used with the LightCycler software to automatically determine the crossing point for individual samples, as previously described. Standard curves were generated by using quadruplicate cDNA standard. Relative standard curves describing the PCR efficiency of selected genes were created and used to perform efficiency-corrected quantification with the LightCycler Relative Quantification software (Roche Diagnostics). Gene expression normalization was performed using a selected housekeeping gene (hypoxanthine phosphoribosyltransferase 1 [*HPRT1*]), and results were expressed as calibrated normalized relative quantity (CNRQ) [17]. *CX3CR1 expression* and *CX3CR1 CNRQ* are equivalent terms.

### Statistical analysis

The descriptive analyses comparing surviving and non-surviving patients at D7 and D28 (all-cause mortality) after ICU admission were completed with the usual appropriate tests, such as the *t* test or Mann-Whitney *U* test for quantitative variables and Pearson’s χ^2^ test for qualitative variables. The Monte Carlo resampling method was used to estimate the *p* value of the χ^2^ test of independence in cases of an expected frequency <5. ICU survival curves were created using the Kaplan-Meier method. Every patient was followed for 28 days in the ICU and after ICU discharge. The results of the log-rank tests were associated with the survival representations. ROC curves were built for CX3CR1 expression levels at D1 and D3 according to D7 and D28 mortality, respectively. Best cut-off values (i.e., maximized sensitivity and specificity) for CX3CR1 mRNA expression prediction of mortality were identified according to the calculation of Youden indexes derived from ROC curve analyses. Univariate and multivariate analyses of mortality outcomes at D7 and D28 were studied with logistic regression. Parameters with a *p* value <0.1 in univariate analyses were kept in multivariate analyses. Parameters with >20 % missing values were not included in multivariate analyses. The 0.632+ bootstrap method was applied to adjust for potential overestimation of diagnostic performance and to estimate internal validity. For each model, 1000 resamplings with replacement were performed. The 95 % CI represents the 2.5th and 97.5th percentiles of estimates obtained in bootstrap resamples. All bootstrap estimations were performed using the ModelGood package in R. The level of significance was set at 5 %, and the results were described with a 95 % CI. The analyses were performed with IBM SPSS Statistics version 20 software (IBM, Armonk, NY, USA).

## Results

### Cohort description

**Fig. 1:**
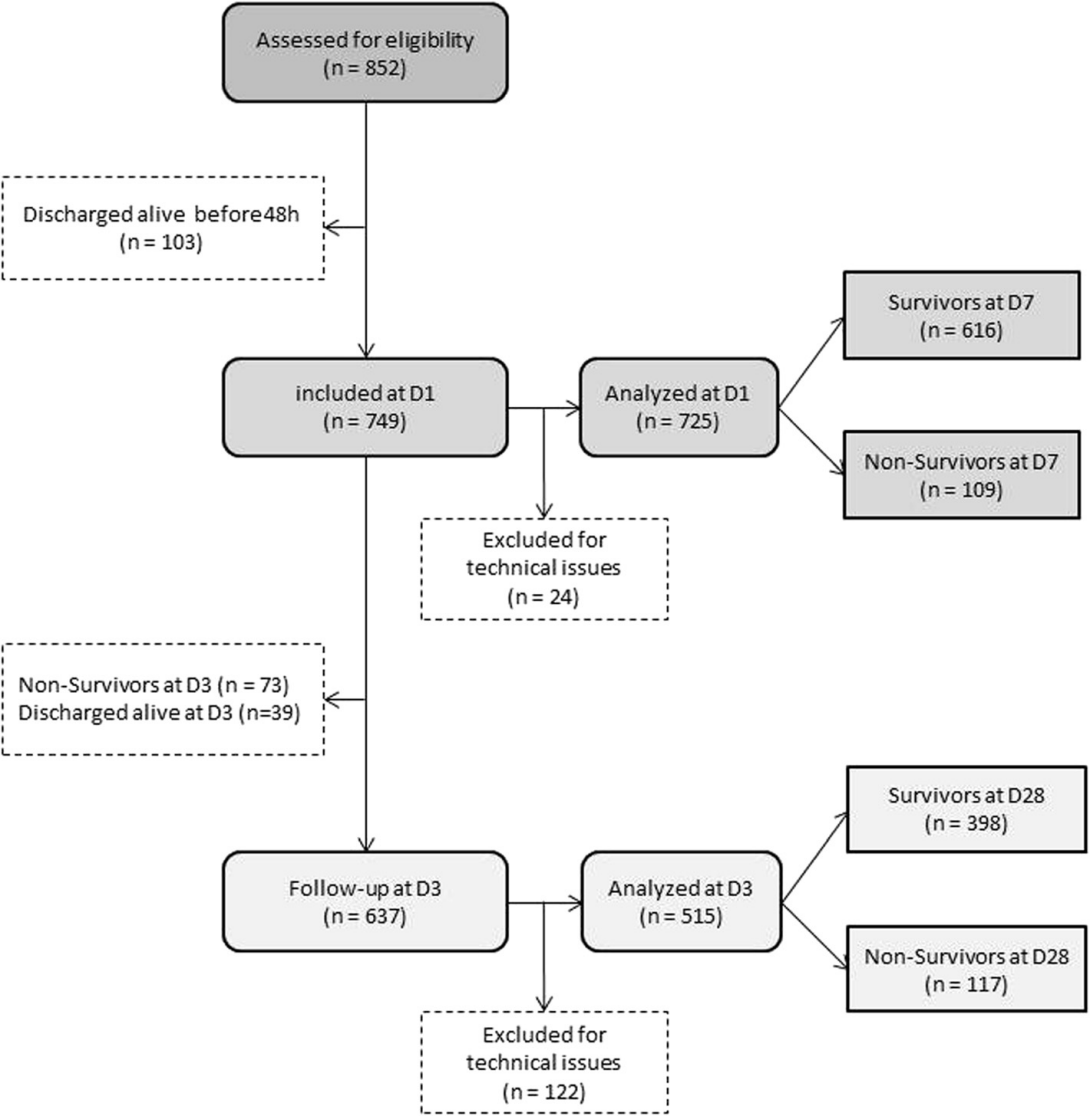
Study flowchart. Between December 2009 and June 2011, a total of 852 critically ill patients were screened for eligibility. After exclusion for technical reasons or intensive care unit discharge within the first 48 h, 725 patients were ultimately included in the study. *D* day

Between December 2009 and June 2011, 852 ICU patients were screened for eligibility. After exclusion due to ICU discharge within the first 48 h, 749 patients were included. Because of technical issues, samples from 24 of these patients could not be processed at D1. Therefore, 725 patients were ultimately analysed (Fig. 1). Their clinical characteristics are described in Table 1. At D3, 515 patients were still alive and had samples available for analysis. The clinical characteristics of this subcohort of patients at admission were not different from those of the overall cohort.

**Table 1:** Clinical and demographic characteristics of critically ill patients in the study

Characteristics	Clinical characteristics at D1			Clinical characteristics at D3		
	Total (*n* = 725)	D7 survivors (*n* = 616)	D7 non-survivors (*n* = 109)	Total (*n* = 515)	D28 survivors (*n* = 398)	D28 Non-survivors (*n* = 117)
Age, years	65 [54–76]	64 [52–75]	71 [62–79]^a^	64 [53–75]	62 [51–74]	73 [63–80]^a^
Male sex	450 (62.1 %)	391 (63.5 %)	59 (54.1 %)	335 (65.0 %)	252 (63.3 %)	83 (70.9 %)
SAPS II	56 [42–69]	53 [40–65]	79 [65–97]^a^	55 [42–67]	52 [39–63]	62 [51–73]^a^
SOFA score at D1	9 [6–12]	8 [6–11]	13 [10–15]^a^	6 [4–10]	6 [3–8]	9 [6–13]^a^
Charlson comorbidity index score	2 [0–3]	2 [0–3]	2 [1–4]	1 [0–3]	1 [0–3]	2 [1–4]^a^
Lactic acid, mmol/L	2.2 [1.4–3.6]	2.0 [1.3–3.1]	4.2 [2.5–8.9]^a^	1.4 [1.1–2.0]	1.3 [1.1–1.8]	1.8 [1.4–2.5]^a^
Respiratory support^b^	581 (80.1 %)	478 (77.6 %)	103 (94.5 %)^a^	402 (78.1 %)	295 (74.1 %)	107 (91.5 %)^a^
Haemodynamic support^c^	489 (67.4 %)	397 (64.4 %)	92 (84.4 %)^a^	231 (44.9 %)	162 (40.7 %)	69 (59.0 %)^a^
Shock at D1^d^	375 (51.7 %)	288 (46.8)	87 (79.8 %)^a^	257 (49.9 %)	193 (48.5 %)	64 (54.7 %)
Shock at D3^d^				121 (23.5 %)	76 (19.1 %)	45 (38.5 %)^a^
Septic shock	279 (38.5 %)	222 (36.0 %)	57 (52.3 %)^e^	192 (37.3 %)	146 (36.7 %)	46 (39.3 %)
Diagnostic category						
Medical	502 (69.2 %)	421 (68.4 %)	81 (74.3 %)	357 (69.3 %)	270 (67.8 %)	87 (74.4 %)
Planned surgery	32 (4.4 %)	31 (5.0 %)	1 (0.9 %)	24 (4.7 %)	18 (4.5 %)	6 (5.1 %)
Urgent surgery	191 (26.4 %)	164 (26.6 %)	27 (24.8 %)	134 (26.0 %)	110 (27.6 %)	24 (20.5 %)
Trauma	67 (9.2 %)	63 (10.2 %)	4 (3.7 %)	57 (11.1 %)	54 (13.6 %)	3 (2.6 %)^e^
Sepsis	506 (69.8 %)	440 (71.4 %)	66 (60.6 %)	364 (70.7 %)	280 (70.3 %)	84 (71.8 %)
Community-acquired	323 (44.6 %)	283 (45.9 %)	40 (36.7 %)	229 (44.5 %)	183 (46.0 %)	46 (39.3 %)
Hospital-acquired	183 (25.2 %)	157 (25.5 %)	26 (23.9 %)	135 (26.2 %)^e^	97 (24.4 %)	38 (32.5 %)
Site of infection						
Respiratory	260 (35.9 %)	229 (37.2 %)	31 (28.4 %)	197 (38.3 %)	149 (37.4 %)	48 (41.0 %)
Abdominal	113 (15.6 %)	90 (14.6 %)	23 (21.1 %)	74 (14.4 %)	50 (12.6 %)	24 (20.5 %)
Others	133 (18.3 %)	121 (19.6 %)	12 (11.0 %)	93 (18.1 %)	81 (20.4 %)	12 (10.3 %)
Type of detection						
Clinical + imaging	124 (24.5 %)	108 (24.5 %)	16 (24.2 %)	96 (18.6 %)	73 (18.3 %)	23 (19.7 %)
Clinical + surgery	23 (4.5 %)	17 (3.9 %)	6 (9.1 %)	14 (2.7 %)	10 (2.5 %)	4 (3.4 %)
Microbiology	339 (67.0 %)	297 (67.5 %)	42 (63.6 %)	240 (46.6 %)	186 (46.7 %)	54 (46.2 %)
Suspected	20 (4.0 %)	18 (4.0 %)	2 (3.1 %)	14 (2.7 %)	11 (2.8 %)	3 (2.6 %)
Identified germ categories						
Gram-negative	196 (57.8 %)	172 (57.9 %)	24 (57.1 %)	134 (55.8 %)	107 (57.5 %)	27 (50.0 %)
Gram-positive	183 (54.0 %)	162 (54.5 %)	21 (50.0 %)	140 (45.6 %)	105 (56.5 %)	35 (64.8 %)
Fungi	8 (2.4 %)	8 (2.7 %)	0 (0.0 %)	6 (2.8 %)	3 (1.8 %)	3 (6.4 %)
Plurimicrobial	124 (36.6 %)	112 (37.7 %)	12 (28.6 %)	95 (39.6 %)	74 (39.8 %)	21 (38.9 %)
Anti-infectious treatment						
Adequacy	487 (96.4 %)	429 (97.5 %)	58 (89.2 %)	356 (97.8 %)	274 (97.9 %)	82 (97.6 %)
To the antibiogram	291 (59.8 %)	258 (60.1 %)	33 (56.9 %)	211 (59.3 %)	163 (59.5 %)	48 (58.5 %)
To the protocol	196 (40.3 %)	171 (39.9 %)	25 (43.1 %)	145 (40.7 %)	111 (40.5 %)	34 (41.5 %)

*D* day, *SAPS II* Simplified Acute Physiology Score II, *SOFA* Sepsis-related Organ Failure Assessment

Results are presented as number and percent or median and interquartile range. Comparisons between survivors and non-survivors were completed with the appropriate usual tests, such as the *t* test or Mann-Whitney *U* test for quantitative variables and Pearson’s χ^2^ or Monte Carlo tests for qualitative variables

^a^ *p* < 0.001

^b^Mechanical ventilation

^c^Catecholamine or dobutamine

^d^Treatment with norepinephrine ≥0.25 μg/kg/minute or epinephrine

^e^ *p* < 0.01

In the total cohort (*n* = 749), 220 patients died within 28 days (D28 mortality 29 %) (Additional file 2: Figure S1). Half of these deaths occurred before D7 (D7 mortality 15 %) and one-third occurred before D3 (D3 mortality 10 %). The median ICU stay was 7 days.

In the D1–D7 period (*n* = 725), non-survivors were significantly older (*p* < 0.001) and presented with significantly higher SAPS II (*p* < 0.001) and SOFA (*p* < 0.001) scores and increased lactacidaemia at D1 (*p* < 0.001). Non-survivors were more frequently in shock at ICU admission (*p* < 0.001) but had infections less frequently than survivors (*p* = 0.023).

In the D3–D28 period in the subgroup of patients alive at D3 (*n* = 515), D28 mortality was 23 % (*n* = 117 non-survivors). Non-survivors were significantly older (*p* < 0.001) and had higher SAPS II (*p* < 0.001) and SOFA (*p* < 0.001) scores and increased lactacidaemia at D3 (*p* < 0.001).

### Primary endpoint

**Fig. 2:**
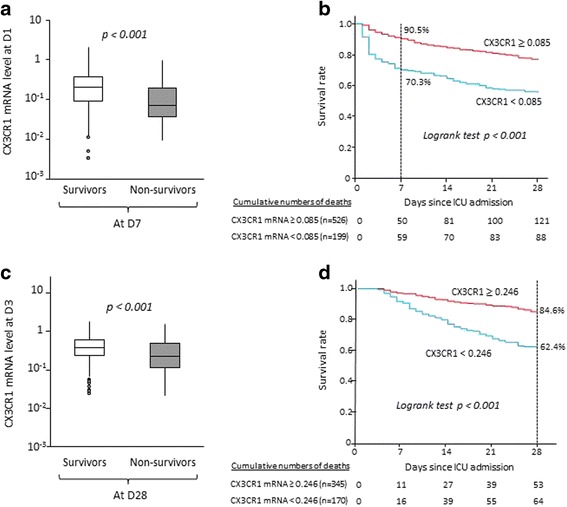
Association between chemokine (C-X3-C motif) receptor 1 (CX3CR1) messenger RNA (mRNA) levels at day 1 (D1) or day 3 and early (day 7) or late (day 28) mortality in critically ill patients. CX3CR1 mRNA levels were measured by quantitative real-time polymerase chain reaction in whole-blood samples of a cohort of 725 adult intensive care unit (ICU) patients. The Mann-Whitney *U* test was used to compare CX3CR1 mRNA levels in survivors versus non-survivors. Kaplan-Meier survival curves were established after stratification based on thresholds defined in ROC curve analyses. A *p* value <0.05 was considered statistically significant. **a** CX3CR1 mRNA levels at D1 in ICU patients were significantly different between D7 survivors and non-survivors. **b** Log-rank test showed that patients with D1 CX3CR1 mRNA levels above the threshold had a significantly better survival than patients with CX3CR1 mRNA levels below the threshold. **c** CX3CR1 mRNA levels at D3 in ICU patients were significantly different between D28 survivors and non-survivors. **d** Log-rank test showed that patients with D3 CX3CR1 mRNA levels above the threshold had a significantly better survival than patients with CX3CR1 mRNA levels below the threshold

At D1, CX3CR1 mRNA expression was significantly lower in non-survivors than in D7 survivors (*p* < 0.0001) (Fig. 2a).

On the basis of ROC curve analysis, a threshold of 0.085 was defined as the best cut-off value for the prediction of D7 mortality (AUC 0.70, 95 % CI 0.65–0.76, sensitivity [Se] 55 %, specificity [Sp] 77 %, positive predictive value [PPV] 30 %, negative predictive value [NPV] 91 %, positive likelihood ratio [LR+] 2.42, negative likelihood ratio [LR−] 0.58). The internal validity of these results was confirmed using a bootstrapping technique (AUC 0.70, 95 % CI 0.63–0.77). Using this threshold, Kaplan-Meier survival curves were established (Fig. 2b). Log-rank test analysis showed that patients with D1 CX3CR1 mRNA expression over the threshold had better survival than patients with CX3CR1 expression below the cut-off (91 % and 70 %, respectively; *p* < 0.001).

In univariate and multivariate logistic regression analyses, low CX3CR1 expression (below threshold of 0.085) remained significantly associated with D7 mortality independently of confounding parameters (adjusted OR [aOR] 2.03, 95 % CI 1.19–3.46, *p* = 0.009) (Table 2). In these models, SAPS II and SOFA scores, presence of sepsis and lactacidaemia at D1 were also associated with D7 mortality. Of note, because the SOFA score was based on norepinephrine or epinephrine administration, a similar multivariate analysis including the presence of shock instead of the SOFA score was performed. In this analysis, neither shock nor Charlson comorbidity index was associated with D7 mortality (Additional file 3: Table S2A).

**Table 2:** Univariate and multivariate analyses of mortality according to CX3CR1 messenger RNA expression in critically ill patients

	Univariate analysis		Multivariate analysis	
Variable	OR (95 % CI)	*p* Value	OR (95 % CI)	*p* Value
D7 mortality and CX3CR1 mRNA expression at D1				
CX3CR1 at D1 < 0.085	4.01 (2.63–6.11)	<0.001	2.03 (1.19–3.46)	0.009
SAPS II	1.08 (1.06–1.09)	<0.001	1.06 (1.04–1.08)	<0.001
Sepsis	0.61 (0.40–0.94)	0.023	0.37 (0.22–0.65)	<0.001
SOFA score at D1	1.35 (1.27–1.44)	<0.001	1.09 (1.01–1.19)	0.047
Lactate at D1^a^	1.27 (1.19–1.34)	<0.001	1.11 (1.03–1.19)	0.005
Charlson comorbidity index	1.08 (0.99–1.18)	0.085	0.99 (0.88–1.10)	0.813
D28 mortality and CX3CR1 mRNA expression at D3				
CX3CR1 at D3 < 0.246	3.33 (2.17–5.10)	<0.001	2.34 (1.45–3.77)	<0.001
Charlson comorbidity index	1.19 (1.09–1.30)	<0.001	1.15 (1.05–1.27)	0.004
SAPS II	1.04 (1.03–1.05)	<0.001	1.02 (1.01–1.04)	0.002
SOFA score at D3	1.20 (1.14–1.26)	<0.001	1.11 (1.05–1.18)	0.001
Lactate at D3^b^	2.12 (1.57–2.86)	<0.001		
Sepsis	1.07 (0.68–1.69)	0.763		

*Abbreviations: D* day, *SAPS II* Simplified Acute Physiology Score II, *SOFA* Sepsis-related Organ Failure Assessment, *CX3CR1* chemokine (C-X3-C motif) receptor 1

Univariate and multivariate analyses on mortality were studied through logistic regressions

^a^33 missing values

^b^140 missing values

At D3, CX3CR1 mRNA expression was lower in patients who died within 28 days after admission (*n* = 117) versus survivors (*n* = 398) (*p* < 0.0001) (Fig. 2c). Based on ROC curve analysis, the best cut-off value for D3 CX3CR1 mRNA expression association with D28 mortality was 0.246 (AUC 0.64, 95 % CI 0.58–0.71, Se 73 %, Sp 56 %, PPV 85 %, NPV 38 %, LR+ 1.65, LR− 0.48). Again, the internal validity of these results was confirmed using a bootstrapping technique (AUC 0.64, 95 % CI 0.56–0.72). Kaplan-Meier survival curves were generated with this threshold. Log-rank test analysis showed that D28 survival was significantly higher in the group of patients with D3 CX3CR1 expression over threshold than in patients with CX3CR1 expression below the cut-off value (85 % versus 62 %, respectively; *p* < 0.001) (Fig. 2d). Finally, in multivariate analysis, low CX3CR1 expression at D3 was associated with increased risk of death at D28, with an aOR of 2.34 (95 % CI 1.45–3.77; *p* < 0.001) (Table 2B). This association was independent of SAPS II and SOFA scores as well as the Charlson comorbidity index. Of note, due to the high number of missing values for lactacidaemia at D3, this parameter was not included in the multivariate analysis. In multivariate analysis, replacement of SOFA score at D3 by presence of shock at D3 did not reveal any significant association between this last parameter and D28 mortality (Additional file 3: Table S2B).

### Secondary endpoint: specificity of septic shock patients

The clinical characteristics of septic shock patients are presented in Table 3.

**Table 3:** Clinical and demographic characteristics of septic shock patients

	At D1 according to survival at D7			At D3 according to survival at D28		
Characteristics	Total (*n* = 279)	Survivors (*n* = 222)	Non-survivors (*n* = 57)	Total (*n* = 192)	Survivors (*n* = 146)	Non-survivors (*n* = 46)
Age, years	67 [58–77]	67 [57–77]	69 [62–77]	67 [57–78]	67 [56–77]	73 [62–79]
Male sex	171 (61.3 %)	140 (63.1 %)	31 (54.4 %)	122 (63.5 %)	91 (62.3 %)	31 (67.4 %)
SAPS II	64 [52–78]	59 [48–71]	88 [70–98]	60 [50–73]	58 [48–70]	68 [57–80]
SOFA score at D1	11 [9–13]	10 [8–12]	14 [12–16]	8 [5–12]	8 [4–11]	12 [8–15]
Charlson comorbidity index score	2 [1–3]	2 [1–3]	2 [1–4]	2 [1–3]	2 [1–3]	3 [1–4]
Lactic acid, mmol/L^a^	2.6 [1.8–5.2]	2.5 [1.7–3.9]	4.8 [2.7–8.8]	1.6 [1.2–2.2]	2.2 [1.6–3.4]	3.1 [1.9–6.1]
Respiratory support^b^	243 (87.1 %)	187 (84.2 %)	56 (98.1 %)	157 (81.8 %)	117 (80.1 %)	40 (87.0 %)
Diagnostic category						
Medical	184 (65.9 %)	144 (64.9 %)	40 (70.2 %)	127 (66.1 %)	98 (67.1 %)	29 (63.0 %)
Planned surgery	10 (3.6 %)	10 (4.5 %)	0 (0.0 %)	6 (3.1 %)	5 (3.4 %)	1 (2.2 %)
Urgent surgery	85 (30.5 %)	68 (30.6 %)	17 (29.8 %)	59 (30.7 %)	43 (29.4 %)	16 (34.8 %)
Type of sepsis acquisition						
Community-acquired	169 (60.6 %)	135 (60.8 %)	34 (59.6 %)	114 (59.4 %)	92 (63.0 %)	22 (47.8 %)
Hospital-acquired	110 (39.4 %)	87 (39.2 %)	23 (40.4 %)	78 (40.6 %)	54 (37.0 %)	24 (52.2 %)
Site of infection						
Respiratory	130 (46.6 %)	107 (48.2 %)	23 (40.4 %)	92 (47.9 %)	72 (49.3 %)	20 (43.5 %)
Abdominal	67 (24.0 %)	49 (22.1 %)	18 (31.6 %)	43 (22.4 %)	27 (18.5 %)	16 (34.8 %)
Others	82 (29.4 %)	66 (29.7 %)	16 (28.0 %)	57 (29.6 %)	47 (32.2 %)	10 (21.7 %)
Type of detection						
Clinical + imaging	55 (19.7 %)	43 (19.4 %)	12 (21.1 %)	39 (20.3 %)	27 (18.5 %)	12 (26.1 %)
Clinical + surgery	15 (5.4 %)	9 (4.1 %)	6 (10.5 %)	8 (4.2 %)	5 (3.4 %)	3 (6.5 %)
Microbiology	198 (71 %)	160 (72.1 %)	38 (66.7 %)	136 (70.8 %)	106 (72.6 %)	30 (65.2 %)
Suspected	11 (3.9 %)	10 (4.5 %)	1 (1.8 %)	9 (4.7 %)	8 (5.5 %)	1 (2.2 %)

*D* day, *SAPS II* Simplified Acute Physiology Score II, *SOFA* Sepsis-related Organ Failure Assessment

Results are presented as number and percent or median and interquartile range

^a^Lactacidaemia: 275 values at D1, 159 values at D3

^b^Mechanical ventilation

**Fig. 3:**
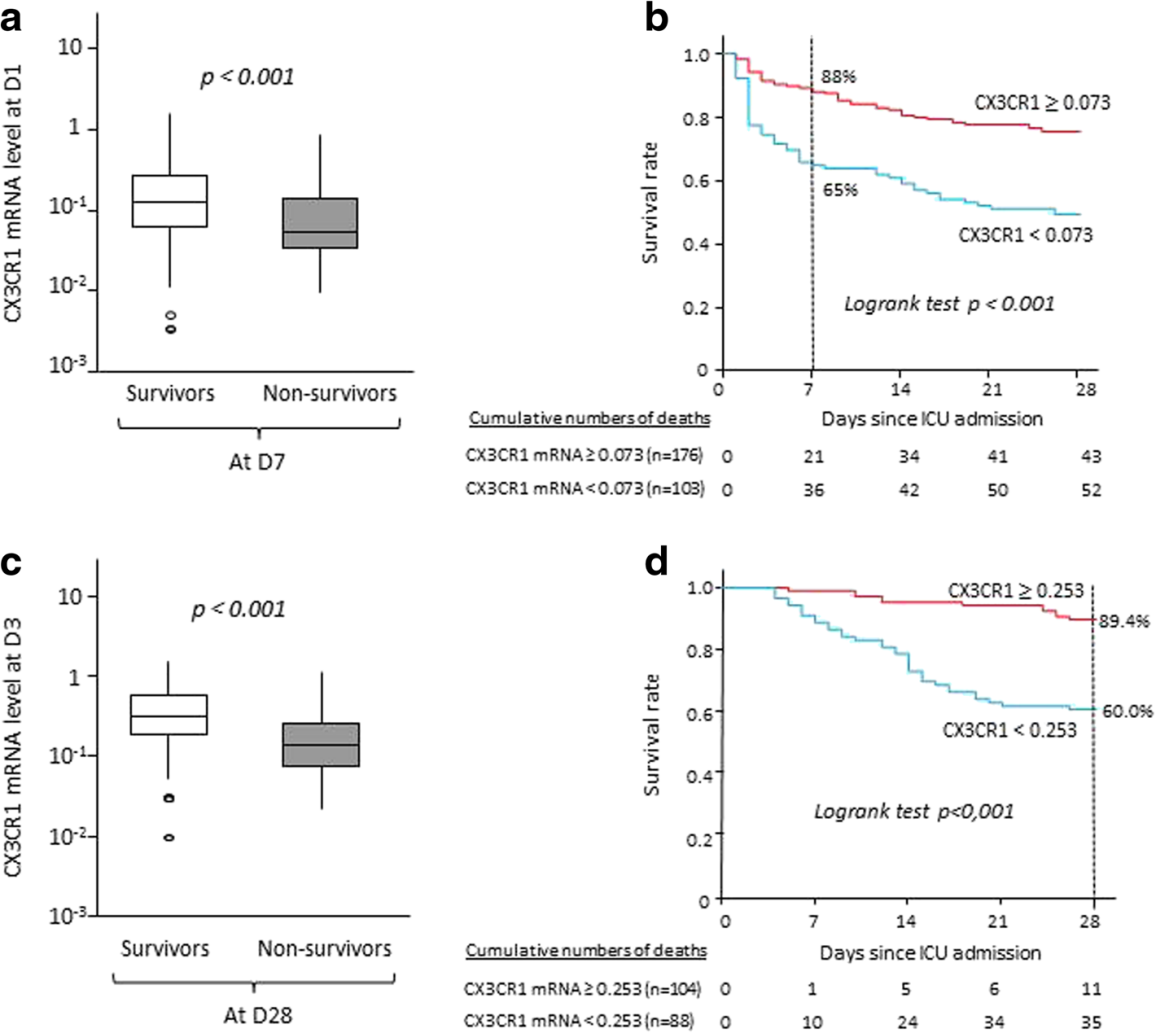
Association between chemokine (C-X3-C motif) receptor 1 (CX3CR1) messenger RNA (mRNA) levels at day 1 (D1) or day 3 and early (day 7) or late (day 28) mortality in septic shock patients. CX3CR1 mRNA level was measured by quantitative real-time polymerase chain reaction in whole-blood samples in a cohort of 279 adult septic shock patients. The Mann-Whitney *U* test was used to compare CX3CR1 mRNA levels in survivors versus non-survivors. Kaplan-Meier survival curves were established after stratification on the basis of thresholds defined in ROC curve analyses. A *p* value <0.05 was considered statistically significant. **a** CX3CR1 mRNA levels at D1 in septic shock patients were significantly different between D7 survivors and non-survivors. **b** Log-rank test showed that patients with D1 CX3CR1 mRNA levels over the threshold had a significantly better survival than patients with CX3CR1 mRNA levels below threshold. **c** CX3CR1 mRNA levels at D3 in septic shock patients were significantly different between D28 survivors and non-survivors. **d** Log-rank test showed that patients with D3 CX3CR1 mRNA levels over the threshold had a significantly better survival than patients with CX3CR1 mRNA levels below the threshold. *ICU* intensive care unit

At D1, CX3CR1 mRNA expression was lower in septic shock patients who died within the first week after ICU admission than in D7 survivors (*p* < 0.0001) (Fig. 3a).

On the basis of ROC curve analysis, a threshold of 0.073 was defined as the best cut-off value for the prediction of D7 mortality (AUC 0.67, 95 % CI 0.59–0.76, Se 63 %, Sp 68 %, PPV 33 %, NPV 88 %, LR+ 1.94, LR− 0.55). Again, the internal validity of these results was confirmed using a bootstrapping technique (AUC 0.67, 95 % CI 0.59–0.76). Using this threshold, Kaplan-Meier survival curves were established (Fig. 3b). Log-rank test analysis showed that patients with CX3CR1 mRNA expression at D1 over the threshold had a better survival than patients with CX3CR1 expression below the cut-off value (88 % and 65 %, respectively; *p* < 0.001).

In univariate and multivariate logistic regression analyses, low CX3CR1 expression (below threshold of 0.073) remained associated with D7 mortality independently of confounding parameters (aOR 2.76, 95 % CI 1.32–5.75) (Table 4). In this model, SAPS II was also associated with D7 mortality, whereas the Charlson comorbidity index score and lactacidaemia did not show any significant association.

**Table 4:** Univariate and multivariate analyses of mortality according to CX3CR1 messenger RNA expression in septic shock patients

	Univariate analysis		Multivariate analysis	
Variable	OR (95 % CI)	*p* Value	OR (95 % CI)	*p* Value
D7 mortality and CX3CR1 mRNA expression at D1				
Charlson comorbidity index score	1.09 (0.96–1.23)	0.205		
CX3CR1 at D1 < 0.073	3.97 (2.16–7.30)	<0.001	2.76 (1.32–5.75)	0.007
SAPS II	1.08 (1.06–1.11)	<0.001	1.07 (1.04–1.09)	<0.001
SOFA score at D1	1.41 (1.26–1.57)	<0.001	1.15 (1.00–1.31)	0.690
Lactate at D1^a^	1.20 (1.12–1.29)	<0.001	1.06 (0.96–1.16)	0.235
D28 mortality and CX3CR1 mRNA expression at D3				
Charlson comorbidity index score	1.15 (1.00–1.33)	0.050	1.16 (0.99–1.36)	0.072
CX3CR1 at D3 < 0.253	5.58 (2.62–11.9)	<0.001	3.98 (1.72–9.23)	0.001
SAPS II	1.03 (1.01–1.05)	0.005	1.02 (0.99–1.04)	0.198
SOFA score at D3	1.20 (1.11–1.30)	<0.001	1.11 (1.01–1.22)	0.031

*D* day, *SAPS II* Simplified Acute Physiology Score II, *SOFA* Sepsis-related Organ Failure Assessment, *CX3CR1* chemokine (C-X3-C motif) receptor 1

Univariate and multivariate analyses of mortality were studied using logistic regression

^a^Four missing values

At D3, 192 septic shock patients were still alive. CX3CR1 mRNA expression was significantly lower in patients who died before D28 than in survivors (Fig. 3c). Based on ROC curve analysis, the best cut-off value for D3 CX3CR1 mRNA association with D28 mortality was 0.253 (AUC 0.72, 95 % CI 0.61–0.81, Se 59 %, Sp 83 %, PPV 92 %, NPV 38 %, LR+ 3.38, LR− 0.50). Again, the internal validity of these results was confirmed using a bootstrapping technique (AUC 0.71, 95 % CI 0.58–0.84). Kaplan-Meier survival curves were generated with this threshold. Log-rank test analysis showed that 28-day survival was higher in the group of septic shock patients with D3 CX3CR1 expression over the threshold than in patients with CX3CR1 expression below the cut-off value (89 % versus 60 %, respectively; *p* < 0.001) (Fig. 3d). In univariate and multivariate analyses, low CX3CR1 expression at D3 was associated with increased risk of death at D28, with an aOR of 3.98 (95 % CI 1.72–9.23; *p* = 0.001) (Table 4). This was independent of SAPS II and SOFA scores as well as the Charlson comorbidity index score.

## Discussion

We demonstrate, for the first time to our knowledge, that decreased CX3CR1 mRNA expression is associated with early and late mortality in critically ill patients independently of initial severity, organ dysfunction, existing comorbidities and metabolic distress. This represents the largest study of such transcriptomic markers’ association with mortality in ICU patients. In addition, this study validates, in a large cohort of patients, our previous results showing that decreased CX3CR1 mRNA is an independent predictor of death after septic shock. This suggests that the monitoring of this host response biomarker in ICU patients may provide information complementary to the usual risk stratification parameters.

Researchers in numerous small observational studies have described the rapid modification of several immune parameters after injury and the association between the extent or duration of these alterations and increased risk of death in these patients [18]. Therefore, in the context of clinical trials already underway in which researchers are evaluating immunoadjuvant therapies in sepsis [19], host response biomarkers are being evaluated as risk stratification parameters in ICU patients. Considering the constantly evolving immune response after severe injury, such biomarkers could be used as a stratification tool or for treatment follow-up [20]. While some markers, such as decreased human leucocyte antigen (HLA)-DR expression on monocytes or lymphopenia, have been proposed as immunomonitoring tools [20], the monitoring of several different markers included in a panel is probably required to accurately assess patients’ immune status after injury [21]. In this context, considering the recent developments of standardized molecular biology techniques, the evaluation of transcriptomic markers presents the advantage of now being fully automated and therefore available with very low pre-analytical constraints [20]. This would most likely facilitate their use on a routine basis, as opposed to markers monitored with less standardized techniques. However, to date, the capacity of such mRNA marker to predict deleterious outcomes in ICU patients has never been evaluated in a large cohort of critically ill patients. In the present study, we evaluated the performance of CX3CR1 mRNA level in the prediction of mortality in a cohort of more than 720 ICU patients. This represents the largest clinical evaluation of a molecular host response marker in ICU patients.

CX3CR1 is a receptor expressed on leucocyte subpopulations. The CX3CR1 ligand (fractalkine) is the only member of the CX3C chemokine subfamily. CX3CR1 has a role in cell chemotaxis to sites of inflammation through interaction with fractalkine fixed on activated endothelial cells. In 2007, Auffray et al. showed that CX3CR1 is overexpressed on “patroller” monocytes and is required for their rapid tissue invasion at the infection site [22]. Interestingly, several studies investigated the expression of CX3CR1 after severe injuries. First, in a microarray study, we identified this gene as the most downregulated gene between survivors and non-survivors of septic shock [9]. This result was confirmed in a subsequent cohort of septic shock patients at both the mRNA and protein levels [10]. Interestingly, decreased CX3CR1 expression on monocytes from septic patients was associated with their decreased activation after ex vivo stimulation. This provides a link between decreased CX3CR1 and immune dysfunction after sepsis. In further studies in murine models of sepsis, investigators proposed a role of this molecule in bacterial killing and host defence against infection as well as in sepsis-induced organ dysfunction [23–26]. This all suggests that measurement of such mRNA markers may help in evaluation of injury-induced immune alterations. Interestingly, besides sepsis, decreased CX3CR1 mRNA expression was also described in sterile inflammation, such as major vascular surgery [27]. Of note, in a recent study of more than 1100 patients, Hoogendijk et al. reported that plasma fractalkine level was positively correlated with severity in patients with sepsis admitted to the ICU [28]. Therefore, the tandem CX3CR1-fractalkine may represent a good prognostic marker in ICU patients. However, the performance of this marker as a risk stratification tool in total ICU patients has not been described yet. Therefore, in the present study, we evaluated the association between CX3CR1 mRNA expression and risk of death in a large cohort of critically ill patients. In addition, we confirmed the association between CX3CR1 mRNA expression and risk of death in the subgroup of septic shock patients.

We chose to evaluate both early (i.e., before D7) and late (i.e., before D28) mortality, hypothesizing that parameters associated with mortality after ICU admission may evolve over time. Indeed, we observed that the clinical parameters associated with mortality were different when we considered early versus late deaths. This was the case both in the total cohort of ICU patients and in the subgroup of septic shock patients. For example, in the multivariate analyses, Charlson comorbidity index, illustrating patients’ comorbidities, was not associated with early mortality but rather with late deaths. Similarly, SOFA score at D1 or the presence of shock at D1 was not associated with early death, while the SOFA score at D3 was significantly associated with D28 mortality. This suggests that the later course of ICU patients with SIRS may be impacted principally by the patient’s medical background (such as comorbidities) and the persistence of organ dysfunction. Conversely, early deaths may be related mostly to the severity of the initial insult. Interestingly, host immune response assessed by CX3CR1 mRNA expression constantly remained associated with both early and late outcomes. This may illustrate that alterations of the host immune response after severe injury may play a role in mortality in the ICU and therefore supports the use of immunoadjuvant therapies in sepsis.

Different CX3CR1 thresholds were selected for prediction of D7 versus D28 mortality. Indeed, cut-off values for the predictive capacity of CX3CR1 at D1 were very low compared with cut-offs at D3. This may illustrate the evolution of the host immune response over time after severe aggression. Severe injury strongly impacts the host immune response by inducing a “genomic storm” [4, 6]. However, in patients who survive this initial aggression, their immune status should return to normal. Failure to return to normal may lead to late mortality [18, 19]. Interestingly, these cut-off values were very similar between ICU patients and the subcohort of septic shock patients. This suggests that underlying pathophysiological mechanisms associated with clinical severity are shared by sterile inflammation and sepsis [4, 12, 29, 30]. In our results, the association between lower CX3CR1 expression and fatal outcome was independent of the presence of either an infection or a shock. Interestingly, non-infectious SIRS seems to be as deleterious as septic shock [31, 32]. In the subgroup of non-infected patients with SIRS, we also observed a significant association between decreased CX3CR1 mRNA level at D1 and D7 mortality (AUC 0.75, 95 % CI 0.66–0.84). In univariate and multivariate logistic regression analyses, low CX3CR1 expression (below threshold of 0.12) in non-infected patients remained significantly associated with D7 mortality independently of confounding parameters (aOR 3.63, 95 % CI 1.43–9.20, *p* = 0.007). However, in this group of patients, no association between D3 CX3CR1 mRNA level and D28 mortality was observed.

This study has some limitations. In particular, we did not include measurement of other markers of injury-induced immunosuppression, such as monocytic HLA-DR. Therefore, we could not compare the performance of our transcriptomic marker versus such a “gold standard” parameter in the present cohort. In addition, as no specific immune function tests were performed, we cannot definitively prove that decreased CX3CR1 mRNA is associated with immune dysfunction in ICU patients. However, considering the interesting results obtained, these two aspects need to be confirmed in a dedicated clinical study.

## Conclusions

We observed that decreased CX3CR1 mRNA expression is a very early phenomenon in critically ill patients and is associated with mortality independently of the presence of shock or sepsis. The recent progress in molecular biology techniques and the development of standardized, fully automated molecular biology platforms should ensure the availability of measurement of this marker around the clock for monitoring of host response in ICU patients. We suggest that the measurement of this marker should be included in a panel of host response markers in forthcoming clinical trials in severely injured patients to identify the group of patients with a high risk of death after initial injury.

## Abbreviations

ACCP/SCCM, American College of Chest Physicians/Society of Critical Care Medicine; aOR, adjusted odds ratio; cDNA, complementary DNA; CNRQ, calibrated normalized relative quantity; CX3CR1, chemokine (C-X3-C motif) receptor 1; D, day; HLA-DR, human leucocyte antigen; ICU, intensive care unit; LR, likelihood ratio; mRNA, messenger RNA; NPV, negative predictive value; PCR, polymerase chain reaction; PPV, positive predictive value; qRT-PCR, quantitative real-time polymerase chain reaction; SAPS II, Simplified Acute Physiology Score II; Se, sensitivity; SIRS, systemic inflammatory response syndrome; SOFA, Sepsis-related Organ Failure Assessment; Sp, specificity

## Additional files

Additional file 1: Table S1. Primer designs. (DOC 29 kb) Additional file 2: Figure S1. Survival curve of the total cohort of intensive care unit patients. (PPT 83 kb) Additional file 3: Table S2. Univariate and multivariate analyses of mortality according to CX3CR1 mRNA expression in critically ill patients. A- D7 mortality and CX3CR1 mRNA expression at D1. B- D28 mortality and CX3CR1 mRNA expression at D3. (DOC 53 kb)
