# Supplementary material for: Decreased CX3CR1 messenger RNA expression is an independent molecular biomarker of early and late mortality in critically ill patients
Source: Crit Care. 2016 Jun 30;20:204. doi: 10.1186/s13054-016-1362-x (PMC4929760; doi:10.1186/s13054-016-1362-x)
Supplement: Additional file 1: Table S1. — Primer designs. (DOC 29 kb) [file 13054_2016_1362_MOESM1_ESM.doc]

**Additional file 1: Table S1. Primer designs.**

Design of primers used for the messenger RNA quantification of the candidate marker (CX3CR1) and housekeeping gene (HPRT1) by quantitative real-time polymerase chain reaction. CX3CR1: chemokine (C-X3-C motif) receptor 1. HPRT1: hypoxanthine phosphoribosyltransferase 1.

| **Gene** | **Accession No.** | **Sequence** |
| --- | --- | --- |
| CX3CR1 | NM_001171174.1  NM_001171171.1  NM_001171172.1  NM_001337.3 | 5‘-CACGCCAGGCCTTCACCATG-3‘ (probe) (20)  5’-AGTCTGAGCAGGACAGGGTG-3’(sense) (20)  5’-GTCCCAAAGACCACGATGTCC-3’(antisense) (21) |
| HPRT1 | NM_000194.2 | 5'-CAAGTTTGTTGTAGGATATGCCC-3' (probe) (23)  5'-CCAAAGATGGTCAAGGTCGC-3' (sense) (20)  5'-GACACAAACATGATTCAAATCC-3' (antisense) (22) |
