# Supplementary material for: Decreased CX3CR1 messenger RNA expression is an independent molecular biomarker of early and late mortality in critically ill patients
Source: Crit Care. 2016 Jun 30;20:204. doi: 10.1186/s13054-016-1362-x (PMC4929760; doi:10.1186/s13054-016-1362-x)
Supplement: Additional file 2: Figure S1. — Survival curve of the total cohort of intensive care unit patients. (PPT 83 kb) [file 13054_2016_1362_MOESM2_ESM.ppt]

## Slide 1
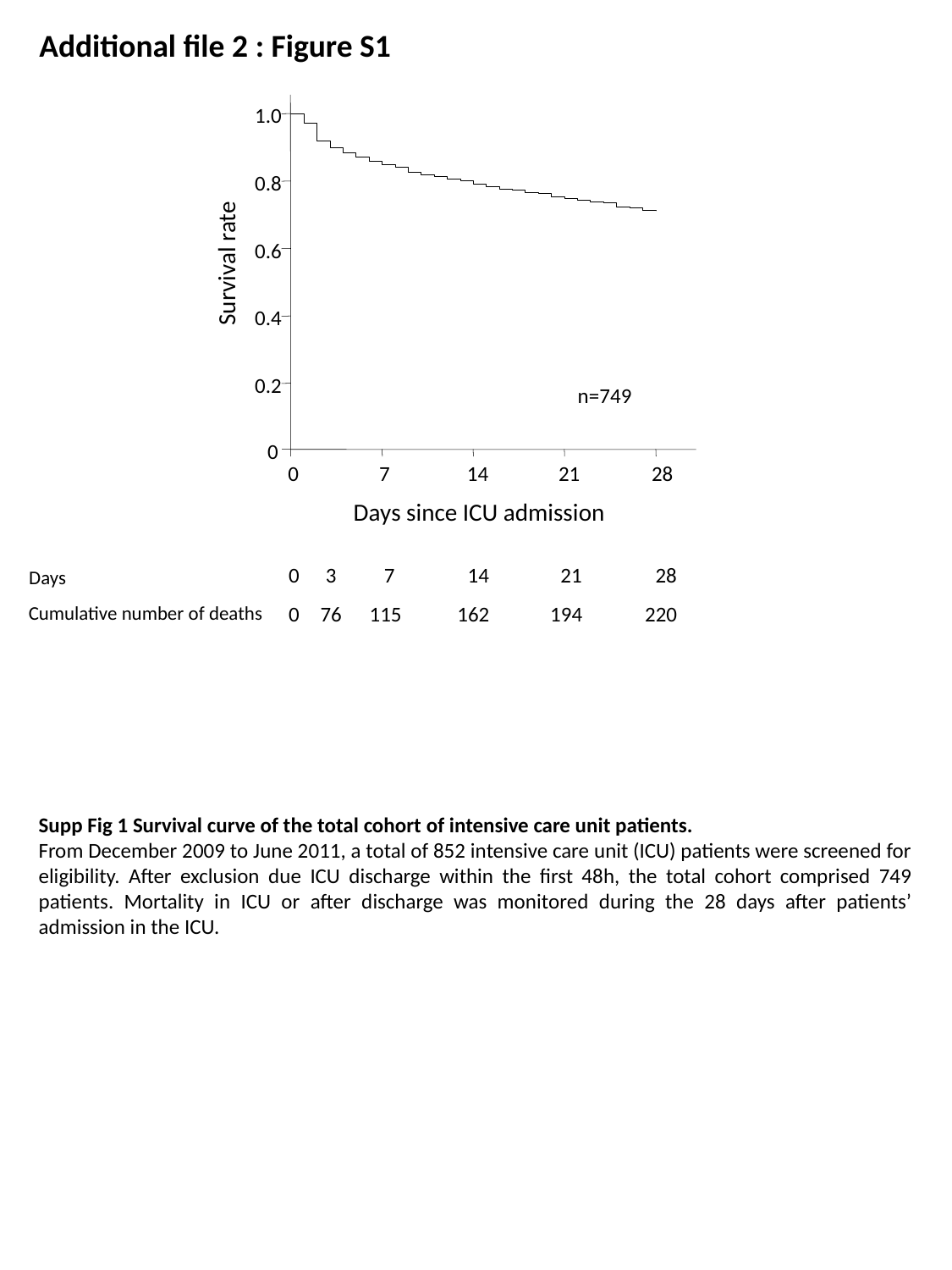

Additional file 2 : Figure S1
1.0
0.8
0.6
Survival rate
0.4
0.2
n=749
0
0
7
14
21
28
Days since ICU admission
3
0
7
14
21
28
Days
Cumulative number of deaths
0
76
115
162
194
220
Supp Fig 1 Survival curve of the total cohort of intensive care unit patients.
From December 2009 to June 2011, a total of 852 intensive care unit (ICU) patients were screened for eligibility. After exclusion due ICU discharge within the first 48h, the total cohort comprised 749 patients. Mortality in ICU or after discharge was monitored during the 28 days after patients’ admission in the ICU.
