# Supplementary material for: Decreased CX3CR1 messenger RNA expression is an independent molecular biomarker of early and late mortality in critically ill patients
Source: Crit Care. 2016 Jun 30;20:204. doi: 10.1186/s13054-016-1362-x (PMC4929760; doi:10.1186/s13054-016-1362-x)
Supplement: Additional file 3: Table S2. — Univariate and multivariate analyses of mortality according to CX3CR1 mRNA expression in critically ill patients. A- D7 mortality and CX3CR1 mRNA expression at D1. B- D28 mortality and CX3CR1 mRNA expression at D3. (DOC 53 kb) [file 13054_2016_1362_MOESM3_ESM.doc]

**Additional file 3: Table S2. Univariate and multivariate analyses of mortality according to CX3CR1 mRNA expression in critically ill patients.** Univariate and multivariate analyses on mortality were studied through logistic regressions. D: Day. SAPSII: Simplified Acute Physiology Score II. SOFA: sepsis-related organ failure assessment score. ICU: Intensive Care Unit. * administration of norepinephrine at 0.25 µg/kg/min and/or epinephrine **33 missing values. ***140 missing values.

1. D7 mortality and CX3CR1 mRNA expression at D1

|  | | | | |
| --- | --- | --- | --- | --- |
|  | **Univariate analysis** | | **Multivariate analysis** | |
| **Variable** | **Odds-Ratio [95% CI]** | **p-value** | **Odds-Ratio [95% CI]** | **p-value** |
| **CX3CR1 at D1 <0.085** | 4.01 [2.63 - 6.11] | <0.001 | 2.11 [1.24 - 3.59] | 0.006 |
| **Charlson** | 1.08 [0.99 - 1.18] | 0.085 | 0.99 [0.89 - 1.11] | 0.919 |
| **SAPSII** | 1.08 [1.06 - 1.09] | <0.001 | 1.07 [1.05 - 1.08] | <0.001 |
| **Sepsis** | 0.61 [0.40 - 0.94] | 0.023 | 0.37 [0.21 - 0.64] | <0.001 |
| **Shock*** | 4.50 [2.75 -7.38] | <0.001 | 1.41 [0.77 - 2.60] | 0.267 |
| **Lactate at D1**** | 1.27 [1.19 - 1.34] | <0.001 | 1.12 [1.05 - 1.20] | <0.001 |

1. **D28 mortality and CX3CR1 mRNA expression at D3**

|  |  |  |  |  |  |  |
| --- | --- | --- | --- | --- | --- | --- |
|  | **Univariate analysis** | | | **Multivariate analysis** | | |
| **Variable** | **Odds-Ratio** **[95% CI]** | | **p-value** | **Odds-Ratio** **[95% CI]** | | **p-value** |
| **CX3CR1 at D3 <0.246** | 3.33 [2.17 - 5.10] | | <0.001 | 2.89 [1.82 - 4.59] | | <0.001 |
| **Charlson** | 1.19 [1.09 - 1.30] | | <0.001 | 1.15 [1.04 - 1.27] | | 0.005 |
| **SAPSII** | 1.04 [1.03 - 1.05] | | <0.001 | 1.03 [1.02 - 1.05] | | <0.001 |
| **Shock at D3*** | 2.65 [1.69 - 4.15] | | <0.001 | 1.34 [0.81 - 2.24] | | 0.259 |
| **Sepsis** | 1.07 [0.68 - 1.69] | | 0.763 |  | | |
| **Lactate at D3***** | 2.12 [1.57 - 2.86] | | <0.001 |  | | |
|  |  |  |  |  |  |  |
